# Respiratory Tract Infections and Laboratory Diagnostic Methods: A Review with A Focus on Syndromic Panel-Based Assays

**DOI:** 10.3390/microorganisms10091856

**Published:** 2022-09-16

**Authors:** Adriana Calderaro, Mirko Buttrini, Benedetta Farina, Sara Montecchini, Flora De Conto, Carlo Chezzi

**Affiliations:** Department of Medicine and Surgery, University of Parma, Viale A. Gramsci 14, 43126 Parma, Italy

**Keywords:** respiratory tract infections, syndromic panels, diagnostic algorithm, time-to-results

## Abstract

Respiratory tract infections (RTIs) are the focus of developments in public health, given their widespread distribution and the high morbidity and mortality rates reported worldwide. The clinical spectrum ranges from asymptomatic or mild infection to severe or fatal disease. Rapidity is required in diagnostics to provide adequate and prompt management of patients. The current algorithm for the laboratory diagnosis of RTIs relies on multiple approaches including gold-standard conventional methods, among which the traditional culture is the most used, and innovative ones such as molecular methods, mostly used to detect viruses and atypical bacteria. The implementation of molecular methods with syndromic panels has the potential to be a powerful decision-making tool for patient management despite requiring appropriate use of the test in different patient populations. Their use radically reduces time-to-results and increases the detection of clinically relevant pathogens compared to conventional methods. Moreover, if implemented wisely and interpreted cautiously, syndromic panels can improve antimicrobial use and patient outcomes, and optimize laboratory workflow. In this review, a narrative overview of the main etiological, clinical, and epidemiological features of RTI is reported, focusing on the laboratory diagnosis and the potentialities of syndromic panels.

## 1. Introduction

Respiratory tract infections (RTIs) are the focus of developments in public health, given their widespread distribution and the high morbidity and mortality rates reported worldwide [1]. The RTIs are defined as diseases of infectious etiology involving the respiratory system [2]. The clinical spectrum ranges from asymptomatic or mild infection to severe or fatal disease, and the severity is the result of the interaction between three factors: the causative agent, the environmental conditions, and the host [1]. These infections typically occur as acute disease with a rapid clinical onset ranging from hours to days after the infection and including a variety of symptoms such as fever, cough, sore throat, coryza, shortness of breath, wheezing, and/or difficulty in breathing [1]. The epidemiology of RTIs is continually evolving following rapid sociodemographic changes and certainly climate change [3,4]. In addition to being the deadliest infectious diseases worldwide, especially among children and elderly, RTIs are the most frequent reason for consultation or admission to health-care facilities and primary care, and they are reported to have a significant impact on the increasing requests for medical examinations at both medical offices and emergency departments, on antimicrobial prescriptions, and on hospitalizations [1,5]. In addition, new epidemiological data highlight the considerable impact of RTIs on the quality and the expectancy of life, as well as the severe threat to populations and global public health [4]. The epidemiological study of RTIs must keep up with the rapid changes in sociodemographic and climate dynamics and needs continuous updating in order to provide important tools for health policies of control and prevention. A prompt and rapid laboratory diagnosis of RTIs is required to support and to guide clinical decisions in favor of appropriate patient management, while also avoiding the inappropriate use of antimicrobials. As a matter of fact, the delay in identifying the causative agent of RTIs could lead to the emergence and spread of antimicrobial-resistant pathogens due to the misuse of broad-spectrum empirical therapy, thus resulting in poor clinical outcomes, increased mortality rates and length of hospital stay [6,7,8].

Important technological advances have been made over the years to provide new tools for the detection of both bacterial and viral respiratory infections, resulting in the development of accurate, fast, and easy-to-use diagnostic methods [9]. In particular, molecular methods are now widely available in diagnostic laboratories. These molecular-based techniques allow sensitive and highly specific detection of both bacterial and viral nucleic acids directly in the clinical specimens and in the cell culture supernatants, without requiring the long incubation period needed for bacterial or viral isolation [9]. In addition, molecular methods involve less technical expertise than culture and are useful for the detection of “difficult to grow” bacteria and of viruses that do not proliferate in standard cell cultures [9].

In this context, the introduction of syndromic panels broke new ground in the field of diagnostic microbiology, since they provide a highly powerful tool capable of detecting a broad array of pathogens that, collectively, could cause a single clinical syndrome; this was achieved by meeting the needs of accuracy and of the shortening of time-to-result [9,10]. In this review, a narrative overview of the main etiological, clinical, and epidemiological features of RTIs is reported, with a focus on the laboratory diagnosis and the potentialities of syndromic panels.

## 2. The Epidemiology of RTIs

RTIs are the deadliest diseases of infectious etiology, and the fourth leading cause of mortality worldwide, with 2,603,913 deaths globally reported in 2019 [4,11].

At present, for the COVID-19 pandemic alone, over 567 million confirmed cases and over 6.3 million deaths have been reported globally [4,11].

In addition, this type of infection is recognized for its significant contribution to loss of life expectancy (LE), with high rates of disability-adjusted life years (DALYs) estimated each year [4,11]. The disease burden of RTIs shows an uneven distribution at both a demographical and geographical level and differs widely by age, gender, and among countries and regions [4]. The negative impact of RTIs on life quality is particularly significant for infants, children, and the elderly, among whom the highest mortality and morbidity rates are also reported, especially in low- and middle-income countries [4,11,12]. Both the pediatric and the elderly populations are shown to be the most vulnerable to RTIs worldwide in terms of mortality and loss of LE. Concerning the pediatric population, the highest mortality and DALY rates are reported among children of less than 1 year [11,12], while among the elderly population, the people over 70 account for the greatest number of deaths and loss of LE. Such disparity in terms of demographic distribution is also observed with regard to the geographic spread of RTIs, largely affected by the degree of socioeconomic development. Low-, and the middle-income countries and territories [13] are more susceptible to RTIs, accounting for the highest mortality and DALY rates [4,11,12]. As concerns high-income countries, where high aging indexes are accounted for, a large number of aged people are at greater risk of infection and hospitalization, resulting in an increasing trend in morbidity, mortality, and loss of LE due to RTIs [4,11,12]. It is worth noting that in high-income countries, many deaths associated with RTIs occur in aged care facilities and in nursing homes; this suggests a high rate of transmission of RTIs in such settings, with reported significant mortality rates and loss of LE for the elderly [4]. Similarly, the pediatric population in high-income countries is at high risk of RTIs due to their attendance at daycare services and schools, which are ideal environments for the transmission of this type of infectious disease.

## 3. The Human Respiratory Tract and the Classification of RTIs

The human respiratory tract is divided into two contiguous spatial environments: the upper tract consisting of the tonsils, nasopharynx, oral cavity, oropharynx, and larynx, and the lower tract which includes the trachea, bronchi, and lungs. Therefore, RTIs are classified as upper respiratory infections (URIs) and lower respiratory infections (LRIs), based on the respiratory tract involved [14]. In this review, the respiratory infections caused by mycobacteria will not be discussed, since mycobacterial infections are not included in the routine laboratory diagnostic workflow and in syndromic panels.

### 3.1. Upper Respiratory Tract Infections (URIs)

**Figure 1:**
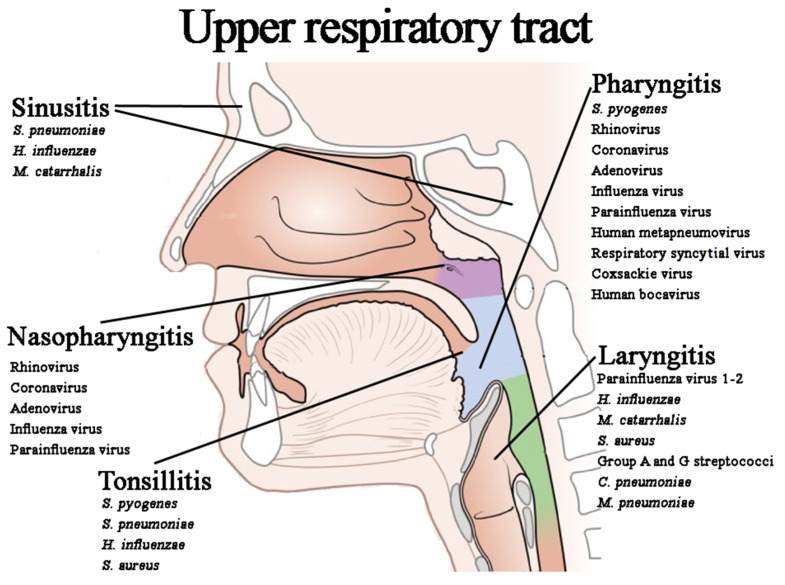
Classification of the URIs with the associated most relevant causative agents.

URIs involve the mucous membranes lining the upper respiratory tract from the nostrils and the mouth to the vocal cords in the larynx, also including the paranasal sinuses and the middle ear [14]. According to the International Statistical Classification of Diseases [15], a URI can occur as acute nasopharyngitis (AN), acute sinusitis (AS), acute pharyngitis (AP), acute tonsillitis (AT), acute laryngitis (AL), and laryngotracheitis or laryngotracheobronchitis (LTB) (Figure 1). The majority of URIs have a viral etiology; however, some of these infections are triggered by bacteria.

#### 3.1.1. Acute Nasopharyngitis (AN)

AN is also known as rhinopharyngitis, acute coryza, or, most commonly, a cold. A cold is inflammation of the nasal and the pharyngeal mucosa mainly caused by infection with rhinovirus (RV) [15,16]. AN is a seasonal infectious disease, particularly spread during the autumn and the winter months, and 90% of cases are due to a viral causative agent. A long stay in indoor crowded environments during the cold season increases the probability of contagion; moreover, most of the respiratory viruses thrive in the low humidity of winter [16]. In addition to RV, Coronavirus (Co-V), Adenovirus (ADV), Influenza (FLU) virus and the Parainfluenza virus (PIV) can cause AN [15,16]. Patients with such infectious diseases complain of cough, pharyngeal pain, a running nose, and a stuffy nose as local symptoms, and increasing fever, general fatigue, and headache as general symptoms [15]. Most of the cases are self-limited and resolve in 7 to 10 days without treatment, although some symptoms last up to three weeks [15,16].

#### 3.1.2. Acute Sinusitis (AS)

AS of infectious etiology occurs as mucosal inflammation of one or more of the paranasal sinuses (maxillary, ethmoid, frontal, and sphenoid) [15].

Similar to AN, the symptoms of infectious AS include nasal congestion and discharge, facial pain over the sinuses, dysosmia, and cough with a mild improvement after 5 to 7 days [15]. The clinical outcome could become worse, with purulent nasal discharge at the middle meatus, olfactory cleavage, maxillary tooth pain, and unilateral maxillary sinus tenderness reported [15,16]. When a worsening of symptoms arises, bacterial etiology is suspected and usually involves *Streptococcus pneumoniae*, *Haemophilus influenzae*, or *Moraxella catarrhalis* [16], whereas *Staphylococcus aureus*, Gram-negative bacilli, *Streptococcus* spp., and anaerobic bacteria are associated more frequently with subacute, chronic, or healthcare-associated sinusitis [17].

#### 3.1.3. Acute Pharyngitis (AP)

AP is defined as inflammation and/or irritation of the mucous membrane of the oropharynx and represents one of the major reasons for outpatient and primary care visit, as well as one of the most common infectious illnesses encountered by general practitioners [16]. Infectious AP especially occurs during the colder months, with peaks of incidence in late winter and early spring, especially among school-aged children and adolescents given the high spread rate of this infectious disease in daycare and schools; adults can be also affected by infectious AP but at lower rates [15,16,18,19,20,21]. Although AP can be caused by many different types of pathogens, most cases have a viral origin [15,16,19,20,21]; in particular, RV and ADV are reported as the primary viral causes of AP, followed by FLU A and FLU B, PIV, Co-V, human metapneumovirus (h-MPV), respiratory syncytial virus (RSV), coxsackievirus, and human bocavirus (h-BocaV). However, cases associated with herpes simplex viruses 1 and 2 (HSV1, 2), Epstein-Barr virus (EBV), human cytomegalovirus (h-CMV), and to the human immunodeficiency virus (HIV) type 1 are also described [18,21,22]. Concerning bacterial etiology, Group B and C β-hemolytic *Streptococcus* spp., *Chlamydia pneumoniae*, *Mycoplasma pneumoniae*, *Candida* spp., mixed anaerobes, *Arcanobacterium haemolyticum*, *Fusobacterium necrophorum*, *Neisseria gonorrhoeae*, and *Corynebacterium diphteriae* are frequently identified as causative agents of AP, but many of the cases are due to *Streptococcus pyogenes* as the leading exponent [18,21,23].

The clinical spectrum of AP includes a broad range of signs and symptoms, which tend to vary depending on the causative agent. Usually, the typical symptoms of AP include discomfort of the throat, throat pain, and swallowing pain, often accompanied with pharyngeal erythema, hyperaemic palatine tonsils, and swelling of the lymphoid follicles of posterior wall of the pharynx [15,21]. If viral in etiology, AP often manifests with coughing, rhinorrhea, conjunctivitis, headache, and rash. When Epstein–Barr virus-associated AP (e.g., infectious mononucleosis) occurs, patients may complain of fever, tonsillar hypertrophy, myalgia, general fatigue, and anterior and posterior lymphadenopathy. Regarding the bacterial origin of the AP, Group A β-hemolytic streptococcal (GAS) pharyngitis is the most prevalent and arises with an acute clinical onset including fever, tonsillar exudates, edematous uvula, and palatine petechiae [16,21]. Viral AP is self-limited, with symptoms lasting from 5 to 7 days, and the clinical course usually resolves without any complication [16]. If not diagnosed and adequately treated, AP can result in serious complications, especially with regard to bacterial cases: untreated GAS pharyngitis can lead to severe sequelae such as peritonsillar abscess, parapharyngeal and retropharyngeal abscess, painful cervical lymphadenitis, sinusitis, otitis media, mastoiditis, sepsis, meningitis, rheumatic fever, poststreptococcal sequelae (i.e., glomerulonephritis), and scarlet fever [16,19].

#### 3.1.4. Acute Tonsillitis (AT)

AT often occurs when an infectious process of the mucosal oropharynx also involves the palatine tonsils, which are bundles of lymphatic tissue located between the palatoglossal arch anteriorly and the palatopharyngeal arch posteriorly [24]. Even though infectious AT usually spreads in winter and early spring, the disease tends to be quite recurrent throughout the year [24].

As well as infectious AP, the etiology of the AT can be either viral or bacterial. Viral AT is quite common and the main causative agents are the same as those of a cold, namely RV, RSV, ADV, and Co-V. On the other hand, although bacterial AT can be caused by different aerobic and/or anaerobic pathogens, most of the cases are due to *Streptococcus pyogenes*, *Staphylococcus aureus*, *Streptococcus pneumoniae*, and *Haemophilus influenzae* [24,25]. Infectious AT usually occurs with swollen tonsils, with associated odynophagia and dysphagia, sore throat, difficulty swallowing and, occasionally, purulent plugs in the tonsillar crypts, high fever, headache, and general fatigue [15,24]. In most viral-origin cases, the prognosis is favorable, and the infectious process resolves spontaneously without requiring hospital admission and/or antimicrobial treatment [25]. Patients with infectious AT commonly recover within a few days without any complications or long-term problems [24]. However, although AT is generally associated with good clinical outcomes, complications can arise when the infection extends to the peritonsillar space, with the subsequent formation of peritonsillar abscesses, especially in cases of a bacterial origin and/or delayed or inadequate antimicrobial therapy [15,24].

#### 3.1.5. Acute Laryngitis (AL) and Laryngotracheobronchitis (LTB)

AL is defined as inflammation of the larynx, resulting in erythema and oedema of the laryngeal mucosa with consequent huskiness or loss of the voice, harsh breathing, dysphonia, and/or a painful dry cough [15,26,27]. Such a clinical feature is one of the most common infectious diseases encountered by primary care physicians, especially among school-aged children, adolescents, and adults, with the same seasonal trend observed for URI [27]. Laryngitis typically occurs with an acute onset because of the spread of viral URIs involving the adjacent structures of the upper respiratory airways, either by directly infecting the laryngeal tissues or by stimulating excessive secretions that lead to inflammation [26,27]. All the major respiratory viruses are etiologically associated with AL; in particular, PIV, RV, FLU, and ADV are the most reported [26,27]. On the contrary, bacterial etiology of AL is rare but cannot be ruled out. In particular, *M. catarrhalis* and *H. influenzae* are the most recovered bacteria in patients with AL, thus suggesting their potential involvement in the pathogenesis of such infectious disease [27]. Before the vaccination era, *C. diphtheriae* was the main bacterial pathogen involved in laryngeal infectious disease. Nowadays, acute laryngitis secondary to diphtheria is rare; however, such cases can occur in unvaccinated populations [26,27]. Other bacterial pathogens identified in patients complaining of symptoms of AL include Group A and G β-hemolytic *Streptococcus* spp., methicillin-resistant *Staphylococcus aureus* (MRSA), *C. pneumoniae*, *M. pneumoniae*, and *Bordetella pertussis*. These two latter pathogens are thought to be especially involved in the pathogenesis of chronic laryngitis in adults [27]. The disease is usually mild and self-limited, and symptoms resolve in an average of 3 days.

Given the crossroad position of the larynx, located between the upper and the lower respiratory system, any infectious disease affecting this anatomical site can easily spread to the surrounding organs, and to the proximal tract of the tracheobronchial tree, also involving its distal portion [26,28]. This condition is referred to as laryngotracheitis or laryngotracheobronchitis (LTB). LTB, more commonly referred to as croup, results from a mucosal inflammation of the subglottic area due to a viral infection of the neighboring anatomical structure [26,28,29]. Such acute disease is an age-specific clinical syndrome since it exclusively affects children between 6 months and 3 years old [28,29]. This pediatric age group is the most prone to the edematous consequences associated to the infection, resulting in the obstruction of the upper respiratory airways, leading to a barking cough, hoarseness, and inspiratory stridor [28,29].

Regarding the etiology, PIV type 1 is the most common viral cause of croup, followed by PIV type 2. Other viruses such as RSV, ADV, and measles (at the onset of measles disease, when mucositis occurs) are a few of the other agents associated with viral croup [26,29]. LTB presents with an acute onset and usually resolves within 2 days in most children [29]. Mucosal damage and the obstruction of the upper airways due to croup are predisposing factors for other infectious diseases such as the bacterial epiglottitis and tracheitis that, unlike the viral processes, occur with a rapid progressive course, high fever, a toxic appearance, and drooling [26,28,29].

Epiglottitis is inflammation of the epiglottis and supraglottic structures characterized by marked swelling of the epiglottic mucosa, and is associated with a high risk of acute and complete airway obstruction, especially in young children [26,28,29]. Before the vaccine introduction, the main causative agent of epiglottitis was *H. influenzae* serotype 1, although *H. influenzae* serotypes A and F and non-typeable strains, *Streptococcus pyogenes* and *Staphylococcus aureus*, were also reported in sporadic cases [26,28,29].

Bacterial tracheitis is an invasive and exudative bacterial infection of the soft tissues of the trachea, resulting in a strikingly rapid onset and progression of the illness, with high fever and a toxic appearance. The main causative agents are to be searched among the inhabitants of the oropharyngeal microbial population such as *Staphylococcus aureus*, and *Streptococcus pyogenes*, or *Streptococcus pneumoniae*, also followed by Gram-negative enteric bacteria such as *Escherichia coli*, *Klebsiella pneumoniae*, and *Pseudomonas aeruginosa* [26,28,29]. The onset of both bacterial epiglottitis and tracheitis mimics that of common and usually benign croup; however, their clinical features could lead to potential life-threatening outcomes [29].

### 3.2. Lower Respiratory Tract Infections (LRIs)

**Figure 2:**
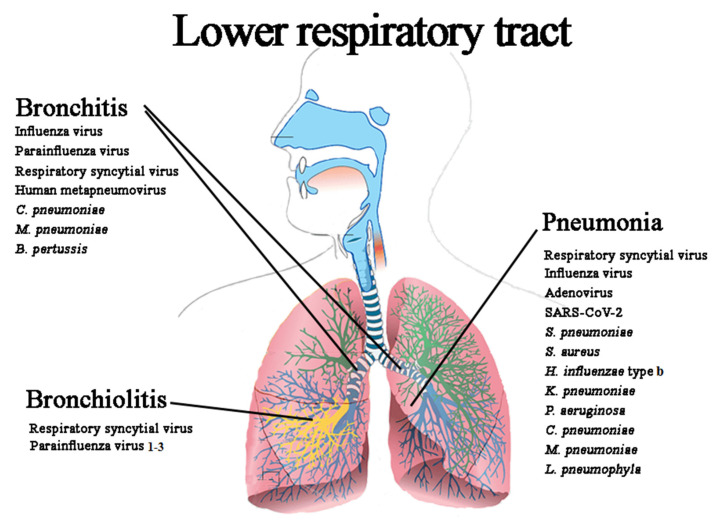
Classification of LRIs with the associated most relevant causative agents.

LRIs are acute infectious illnesses involving the bronchi, bronchioles, alveoli, and lungs. The term LRIs is a broad definition that refers to a variety of infectious inflammatory diseases of the lower respiratory airways, among which acute bronchitis (AB), acute bronchiolitis (ABR) and pneumonia are major matters of concern (Figure 2).

#### 3.2.1. Acute Bronchitis (AB)

AB is defined as brief, self-limited inflammation in response to an infectious process that involves mucosa lining the large and mid-sized airways, mainly resulting in acute cough with or without sputum production [15,30,31]. Although it is a recurrent year-round clinical syndrome, AB mostly occurs during the cold. This infectious disease is primarily caused by a viral infection, with variable rates of prevalence according to the epidemiology of the viral pathogen involved [30,31,32,33]. The main viruses identified as leading viral causes of AB include FLU A and B, PIV, RSV, and h-MPV, as well as common upper respiratory viruses, such as RV, Co-V, and ADV [30,31]. In particular, FLU A and B viruses are responsible for winter outbreaks of AB in both children and adults because of their high rates of transmission during the cold months and their efficiency in infecting and damaging the bronchiolar epithelial cells [30,31]. Approximately 10% or fewer of the AB cases are referred to atypical bacteria, especially *C. pneumoniae*, *M. pneumoniae*, and *Bordetella pertussis* [30,31]. These latter two are associated with more severe cases of AB with long periods of incubation. Although bacterial species are rarely associated with AB, there is wide evidence of their key role in the pathogenesis of acute exacerbations of chronic bronchitis (AECB), a different clinical syndrome caused by multiple factors such as environmental exposure, infections, inflammation, and genetic predisposition [34]. *S. pneumoniae*, *H. influenzae*, and *M. catarrhalis* represent the main colonizing bacteria of the lower airways in AECB, with local findings of *P. aeruginosa, Stenotrophomonas maltophilia*, and *Enterobacteriaceae* in patients with a high degree of functional pulmonary impairment [34].

The clinical course and the severity of the symptoms associated with AB vary according to the causative agent; in mild cases, the illness lasts from 7 to 10 days, whereas more severe cases persist for up to 3 weeks [30,31].

#### 3.2.2. Acute Bronchiolitis (ABR)

ABR occurs as infection-induced inflammation of the respiratory epithelium lining the bronchioles, resulting in the obstruction of these smaller airways and consequent wheezing commonly associated with fever, cough, rhinorrhea, dyspnea, and tachypnea [15,26,29]. This clinical syndrome is age-specific, since it typically affects children younger than 2 years, with an incidence peak occurring between 2 and 6 months of age [26,29]. With regard to epidemiology, ABR has a yearly seasonal pattern that varies according to the geography, the climate, and the causative agent [29]. The recognized causative agents are only viruses, with RSV identified as the major causative pathogen [33,35]. RSV represents the principal agent in two thirds of the cases of bronchiolitis, with high rates encountered in hospitalized patients: RSV-associated diseases have caused an estimated 1.8 million hospital admissions and 40,000 deaths among children [26,29,33,35]. In addition, RSV is the leading cause of hospitalization for ABR in the first year of life [29,33,35]. Other viruses may play a role in the pathogenesis of ABR, including h-MPV, RV, FLU, PIV serotypes 1–3, ADV, h-BocaV, and Co-V (in particular, NL63, HKU1, 229E, and OC43 species) and they are usually involved as coinfecting agents [29,33,35]. An acute course of ABR usually lasts from 3 to 7 days. A minority of children complain of severe symptoms such as hypoxemia, apnea, or respiratory failure and require admission to intensive care. In most cases, the clinical conditions of the hospitalized children with ABR tend to improve within 3 to 4 days with a median 2-week recovery period [26,29].

#### 3.2.3. Pneumonia

Pneumonia is an acute infection of the pulmonary parenchyma causing mild to severe illness in people of all ages [36].

Among all the infectious diseases affecting the respiratory system, pneumonia has the greatest impact on public health since it remains a leading cause of hospitalization and death worldwide. In particular, higher rates of mortality due to pneumonia are reported in children, among whom the disease accounts 14% of all deaths of children under five years old, and 22% of all deaths in children aged 1 to 5 [36]. Pneumonia affects children and families worldwide, but the mortality rates are highest in South Asia and Sub-Saharan Africa [36].

Two types of pneumonia are recognized based on both their clinical presentation and their etiology. The most frequent is typical pneumonia caused by pyogenic bacteria (typically *S. pneumoniae*) and currently named bacterial pneumonia; this presents with the typical symptoms including hyperpyrexia (>38.5 °C), a productive cough and general malaise. The other type is interstitial pneumonia, mainly caused by viruses and atypical bacteria (i.e., RSV, *Legionella*) and presenting with poor symptoms such as a dry and irritating cough and mild fever (no more than 38 °C). Chest imaging of typical pneumonia reveals the obstruction of alveoli by purulent material, limiting the space; it often involves a pulmonary lobe, and a ground-glass picture in cases of interstitial pneumonia, due to viruses evolving until typical alveolar obstruction in the case of legionnaires’ diseases by *Legionella pneumophila* [37].

The most common categories of pneumonia include community-acquired pneumonia (CAP) and hospital-acquired pneumonia (HAP). CAP is due to an infection acquired outside of the hospital setting, while HAP occurs among intubated patients after at least 48 h of hospitalization [38]. Moreover, HAP includes two minor subcategories known as ventilator-associated pneumonia (VAP) and healthcare-associated pneumonia (HCAP) [38]. VAP involves patients receiving mechanical ventilation and symptoms with a 48–72 h incubation time-period after endotracheal intubation [38]. HCAP frequently spreads in lower-acuity health care settings such as nursing homes and dialysis centers [38]. Hemorrhagic alveolitis pneumonia due to *Pneumocystis jirovecii* is also reported in immunocompromised patients including those with HIV infection [36].

A wide variety of agents, including bacteria, viruses, and fungi, can avoid or overwhelm the immune defenses of both the upper respiratory and the lower respiratory tract (Table 1), thus colonizing the parenchyma of the lungs and triggering the infectious process. If bacterial in etiology, the pathogenesis mainly involves the lung parenchyma and the alveoli, resulting in the clinical spectrum of typical pneumonia. On the contrary, when the infectious process affects the extra-parenchymal pulmonary interstitial tissue, interstitial pneumonia occurs and it is usually due to viruses (i.e., h-CMV, FLU A, and RSV), and rarely to bacteria such as *Legionella* spp., *M. pneumoniae*, and *C. pneumoniae.*

Regarding the bacterial etiology, *S. pneumoniae* is certainly the leading causative pathogen, accounting for more than 25% of community-acquired pneumonia cases worldwide and the most common cause of bacterial pneumonia in children [36,38,39]. Pneumococcal pneumonia is the most common CAP [40]. *S. aureus* is frequently isolated from patients with HAP, HCAP, and VAP with the major rates accounted for in intensive care units [38,40]. In particular, the impairment of host defenses in hospitalized patients represents a predisposing factor to the colonization of the oropharynx by *S. aureus*, thus contributing to the development of a *S. aureus*-associated pneumonia [40]. In certain cases, pneumonia due to *S. aureus* results from a complication of the widespread dissemination of staphylococcal microorganisms through the bloodstream [40]. The Gram-negative bacteria may also be involved in the pathogenesis of pneumonia, especially, *K. pneumoniae*, *P. aeruginosa*, and *H. influenzae* [38,40]. This latter, in particular, *H. influenzae* type b (Hib), is reported as the second most common cause of bacterial pneumonia [36]. It is worth noting that Gram-negative-bacteria-associated pneumonia normally occurs in the context of hospitalization, a stay in a chronic care facility, the presence of co-morbidities, compromised host defenses, and recent antibiotic therapy [38,39,40]. Moreover, these predisposing factors contribute to the development of infectious processes carried by multidrug-resistant bacteria such as methicillin-resistant *S. aureus* (MRSA) and extended-spectrum β-lactamase (ESBL)-producing *Enterobacteriaceae* [38]. The range of bacteria able to cause pneumonia also includes the anaerobic and the aerobic inhabitants of the microbial population of the oropharynx [40]. Such microorganisms may potentially lead to pneumonia as a consequence of the aspiration of oropharyngeal secretions into the tracheobronchial tree [40]. Patients who are bedridden with impaired consciousness or those with difficulty swallowing are at major risk of developing pneumonia due to such opportunistic pathogens [38,40].

The list of causative bacterial agents of pneumonia also includes obligate intracellular bacteria such as *Legionella pneumophila*, *C. pneumoniae*, and *M. pneumoniae* which are mainly responsible for epidemic and sporadic cases [38,40]. Viruses are also a common cause of pneumonia, especially in hospital settings, in immunocompromised patients and in the elderly. RSV has always been reported as the main viral cause of pneumonia [36], followed by FLU A and ADV [40], until the emergence of SARS-CoV-1, Middle East Respiratory Syndrome (MERS), and the novel SARS-CoV-2 in 2019; the latter is the etiological agent of the present Co-V disease (COVID 19), declared a pandemic by the WHO in March 2020. Before this date, viruses as a cause of frank pneumonia were diagnosed relatively infrequently, except in children. However, the emergence of SARS-CoV-2 certainly contributed to increased rates of viral pneumonia cases, since researchers have established its key role in the pathogenesis of interstitial pneumonia. The rate of interstitial pneumonia was significantly higher during the COVID-19 period (7.1%) compared with that found in the pre pandemic periods (5.15%) (*p* < 0.001) [41].

It should be noted that the finding of a virus does not mean its involvement as a cause of pneumonia, since the disease could also occur as a result of viral infection and secondary bacterial coinfection [38]. The clinical severity of pneumonia is partially attributed to the etiological agent involved: the milder cases are commonly associated with *S. pneumoniae*, *M. pneumoniae*, *C. pneumoniae*, influenza virus, and ADV, whereas the most severe presentations usually involve *S. aureus*, *L. pneumophila*, and *H. influenzae* [38].

Distinguishing between bacterial pneumonia and viral pneumonia is of great importance, especially to avoid unnecessary antibiotic treatment. A diagnosis can be difficult to make with limited technical resources [8], and a combination of laboratory methods is mandatory to achieve the correct diagnosis and appropriate patient management with the administration of prompt targeted therapy; this is of great importance considering that typical pneumonia could evolve into sepsis and meningitis, both correlated with high mortality, and interstitial pneumonia could cause rapid onset respiratory failure and death [37].

## 4. Laboratory Diagnosis

Early and accurate diagnosis of an RTI is crucial for the adequate management of the patient in terms of the appropriate antiviral or antibacterial therapy, effective infection control measures, and the reduction of the hospital stay’s length [42]. Moreover, the laboratory diagnosis must include both microbiological and virological methods to be significantly informative in terms of outbreak management, epidemiological surveillance, antimicrobial susceptibility, and strain typing [43]. Despite the key role of the clinical laboratory, the microbiological/virological diagnosis of RTIs is still challenging given the complexity of such infections [44]. The quality and the diversity of the respiratory specimens, the difficult accessibility to certain anatomical respiratory structures, potential interferences due to the oropharyngeal microbial population, the wide variety of the respiratory pathogens, and the complex pathophysiology of the RTIs are a few of the considerable challenges to the differential diagnosis of these pathogens [42,44].

The diagnosis of RTIs primarily involves preliminary examination of the associated symptoms and signs, in order to define the key clinical question necessary to allow the clinical microbiologist to establish an adequate diagnostic workflow to be undertaken, starting from the selection of the appropriate respiratory specimen [43]. The collection, the transport, the storage, and the processing of the respiratory specimen is crucial for the reliability of the diagnostic results; therefore, physicians and laboratory workers should meticulously follow the reference guidelines to ensure the proper management of the sample [9,17,43].

The diagnostic workflow of RTIs historically relies on many tools to determine the microbial and viral etiology of these infections, such as microscopic examination, conventional culture, traditional cell cultures, antigen detection, and serology [8,42,43]. The implementation of new analytical approaches such as molecular methods [9] allows researchers to broadly maximize the direct detection of respiratory pathogens, especially those hardly detectable and for which the conventional culture is not a feasible identification method [43]. In addition, clinical microbiologists are currently experiencing new significant innovation in the field of molecular diagnostic approaches, such as syndromic panels [45]. In particular, respiratory syndromic panel-based assays allow the simultaneous detection and identification of multiple pathogens associated with the most severe respiratory syndromes [45].

The spectrum of available diagnostic methods for viral and microbial diagnosis is wide, and the knowledge of their associated advantages, limitations, and time-to-results is crucial to better interpret the results and to appropriately integrate the findings into their clinical management [9].

### 4.1. Specimen Collection

The detection of respiratory pathogens largely depends on several preanalytical variables and, certainly, on the type and the quality of the respiratory specimen. In particular, proper specimen management significantly impacts the laboratory diagnosis and the therapeutic decisions, the antibiotic stewardship, the hospital and laboratory costs, the patient care, the clinical outcomes, and the length of hospitalization; moreover, it drives the efficiency of the laboratory [17]. The timing of collection is the first essential condition to ensure accurate microbiological diagnosis and interpretability of the results [43,46]. According to the guidelines, specimens should be collected as early as possible in the acute stage of an infection, preferably prior to the administration of antimicrobial or antiviral drugs [17,43,46]. The respiratory specimens should be collected within 3 days of symptom onset and no later than 7 days, since the viral titer and the amount of bacteria tend to markedly diminish after 72 h from clinical onset [47].

The mode of transportation and the storage of the sample are crucial to preserve both the microbial and the viral characteristics of the specimen [9,43]. The samples should be delivered as quickly as possible to the laboratory. If the respiratory sample cannot be transported to the laboratory or processed within 1–2 h, the guidelines recommend its storage at −80 °C to −20 °C in order to preserve microbial community composition. Whenever this is not possible, the samples should be stored at 4 °C to 8 °C and processed the same day or the following day. It could also be possible to collect the specimens in specific collection tubes containing a preservation transport medium: if these collection tools are available, the sample could be stored for 24 h at room temperature or at 4 °C [48,49]. It is worth noting that the specimens for virus detection should be transported in suitable transport medium tubes [32] on wet ice at 2 °C to 8 °C and frozen at −80 °C if testing is delayed by >48 h [9,17]. On the basis of the suspected etiology, either bacterial or viral, the diagnosis of respiratory tract infections requires a specific type of specimen and collection method, as well as specific transport and storage conditions to optimize the diagnostic yield [9,17].

Although various respiratory specimens can be used for identifying the microbial and viral etiology of an RTI [43,46], only a few types are easily obtainable and recommended in terms of diagnostic utility [17,43].

With regard to URIs, their diagnosis is mostly based on the evaluation of the symptoms and the signs reported by the patient [8,17,43]. Although the diagnosis of a URI is mostly clinical, the guidelines recommend local microbiological sampling whenever a clinical impairment of the infection occurs or when the patient reports signs and symptoms attributable to AP [8,17]. When the laboratory diagnosis of a URI is required, the sampling tools recommended are nasopharyngeal washes, nasopharyngeal aspirates, nasopharyngeal swabs, oropharyngeal swabs, and combined nasopharyngeal and oropharyngeal swabs [17,50]. The nasopharyngeal aspirate and the nasopharyngeal wash are the specimens of choice for the detection of respiratory viruses, since large numbers of respiratory epithelial cells are aspirated during the collection process [17,43,50]. However, the collection of nasopharyngeal aspirates or the nasal washes is hardly feasible for widespread use in clinical practice, since it requires specific suction devices and skilled operators to obtain the specimens [43]. On the contrary, the collection of nasopharyngeal or oropharyngeal swabs is easier and painless and can also be performed outside the hospital setting. A range of commercial swabs are now available, including rayon-tipped swabs, polyester-tipped swabs (Dacron), and polyurethane sponges with wooden, plastic, or wire shafts [43].

When a viral URI is suspected, the clinical samples are usually collected using a Dacron swab and placed in a viral transport medium which contains antibiotics, a buffered salt solution, a proteinaceous substance (such as albumin, gelatin, or serum), and a pH indicator [9]. On the other hand, when a bacterial URI is suspected, Dacron or rayon swabs should not be the tool of choice for oropharyngeal sampling, since they hold small volumes of the sample (0.05 mL), with microbes harnessed within their fibers, thus affecting specimen collection in terms of quality and microbial quantity [17]. The flocked nylon swab is the most valuable tool for respiratory specimen collection, especially for the bacterial diagnosis of a URI, since it allows more efficient release of respiratory epithelial cells and oropharyngeal secretions [17,43]. In particular, the flocked nylon swab makes it easier to obtain bacteria and/or fungi on the solid media and allows a more homogeneous inoculum of the specimen on the agar plate [17].

The range of specimens available from the lower respiratory tract includes spontaneous, or less appropriately, induced sputum; bronchoscopy specimens; endotracheal aspirates; and, quite rarely, transthoracic lung aspiration. Given the expertise and technical skills required and the equipment needed, the collection of specimens other than sputum from the lower respiratory tract may be limited to clinically severe cases including hospitalized patients and life-threatening cases [47]. The collection of lower respiratory specimens is challenging given the “background noise” due to the commensal microbiota of the oropharynx, which could contaminate the specimen during the sampling, thus interfering with the interpretation of the results. For this reason, specimens from the lower respiratory tract require particular care during collection [9,17], and invasive techniques represent efficient and mostly sterile alternatives for pathogen identification. In terms of sterile techniques, bronchoalveolar lavages (BAL) is the most used [8].

### 4.2. Microscopy

Since lower respiratory specimens are likely to be contaminated during collection, microscopy represents a useful tool for assessing the quality of a sample before the culture, in order to overcome potential misinterpretations of the results [43,46]. Moreover, microscopy provides early and concise information about the infection, such as the presence of large numbers of polymorphonuclear (PMN) cells as markers of the inflammatory response, or the presence of bacteria with characteristic morphology [43,46]. The results of microscopic examination may provide early indication of the culture results and give guidance about treatment [43,46].

With regard to microbial URIs, microscopy following the Gram staining of upper respiratory specimens is useful for the detection of PMN cells and some characteristic bacteria such as *C. diphtheriae* and *B. pertussis*, especially in nasopharyngeal aspirate. Generally, Gram staining is not recommended as a reliable tool for the detection of other bacteria (such as streptococci causing pharyngotonsillitis or *N. meningitidis* in healthy carriers) since these cannot be distinguished from the nonpathogenic colonizers of the normal microbial population of the upper respiratory system [50]. Other staining methods such as Loeffler’s Methylene blue for *C. diphtheriae* can be used when specific clinical suspicion is reported to the laboratory [50]. When *P. jirovecii*-associated pneumonia is suspected, the gold-standard staining techniques recommended are direct or indirect immunofluorescence assays, which are proven to be highly sensitive and specific for different life stages, depending on the antibody used [51].

Gram staining and microscopic examination of the sample from a patient complaining of LRI is highly recommended for evaluating the suitability of the specimen. The quality of a lower respiratory specimen is especially evaluated by assessing the number of squamous epithelial cells (SECs) and PMN cells in a Gram-stained smear of the specimen [43,46]. In particular, the presence of a low number of SECs and a high number of PMN cells per low-power field are indicative of a high-quality specimen; on the contrary, specimens with relatively low numbers of PMN cells and high numbers of SECs are likely to represent oropharyngeal contamination and are recommended to be rejected for conventional culture [43,46]. For example, a number of SECs/100× objective microscopic field > 10 shows that the sputum sample contains saliva and is unsuitable; similarly, the presence of >1% SECs indicates contamination from the commensal microbiota of the upper respiratory tract and the sample is considered unacceptable. The specimens of the lower respiratory tract are also examined for inflammatory cells, the presence of bacteria and their characteristics, such as how they Gram stain, their shape, their layout, their number, and their intracellular or extracellular position, and the prevalence of a single microbial population [50]. The stained smears obtained from patients with aspiration pneumonia are characterized by many polymorphonuclear neutrophils and mixed intracellular respiratory flora (commonly streptococci and anaerobes), and should be discriminated from the contaminating respiratory microbiota. The presence of intracellular microbes in alveolar macrophages detected in BAL has high sensitivity and specificity for the diagnosis of VAP. On the basis of the Gram staining, bacteria sharing similar features to the most common respiratory bacterial pathogens should be considered in the interpretation of results, and their presence should be notified to clinicians to guide them for potential empirical therapy. On the contrary, if bacteria are insufficient in quantity or do not show Gram-staining characteristics attributable to a potential pathogen, they should be reported as normal respiratory flora [50].

Microscopy has also been a very important tool in the field of viral RTIs. In particular, electron microscopy has played a key role, even in recent times, in identifying novel viral strains causing epidemics such as, in the early 2000s, the first human Co-V-associated with the Severe Acute Respiratory Syndrome (SARS) [42,52,53]. However, despite its several advantages, the use of electron microscopy in the diagnosis of viral respiratory infections has some limitations: it is laborious, time-consuming, and requires considerable technical skill for accurate analysis, as well as strict control of experimental conditions and a high concentration of viral particles (>10^5^ mL), with a turnaround time ranging from 3 to 16 h (including specimen preparation) [42,54,55]. For these reasons, electron microscopy directly applied to clinical samples is not recommended as a routine diagnostic method for respiratory infections, but rather, for the identification of viruses causing a cytopathic effect after virus cultivation [9].

### 4.3. Culture

Bacterial culture remains, at present, the gold-standard method for the isolation and detection of respiratory pathogens of the higher and lower respiratory tract, including atypical bacteria. However, it is considered a labor-intensive method that requires considerable technical expertise and long time-to-result. In addition, the reliability of such a method is not always guaranteed since it widely depends on the quality of the specimen, which suffers from the contamination that potentially occurs during sampling. Moreover, the culture results could be misinterpreted, especially when specimens are collected after starting antibiotic therapy. The growth of bacterial colonies is followed by the identification of the same ones using biochemical tests or, more recently, using matrix-assisted laser desorption/ionization time-of-flight (MALDI-TOF) mass spectrometry and antimicrobial susceptibility testing (AST) via several manual or automated methods, with a turnaround time of 48–96 h [42]. For these reasons, culture-based identification of a pathogen cannot be considered adequate for allowing a prompt diagnosis and targeted antibiotic therapy, which is required for optimal patient management [42].

With regard to URIs, pharyngeal samples are routinely cultured for *Streptococcus pyogenes* on 5% sheep blood agar or Group A *Streptococcus* selective blood agar (which is easier to visualize because it inhibits accompanying flora but delays the appearance of colonies), and the plates are checked for β-hemolytic colonies. Several other pathogens may cause pharyngotonsillitis or may colonize the upper respiratory tract without causing disease, and their isolation may be important in patients with ear, nose, and throat disorders [50]. Nasopharyngeal specimens are useful for the diagnosis of infection by *B. pertussis*, *C. diphtheriae*, and *Chlamydophila* spp., and moreover, for the detection of *N. meningitidis*, *S. aureus*, and *S. pyogenes* carriages. Such samples are usually inoculated on sheep blood agar or chocolate agar; then, they are aerobically incubated at 37 °C, in 5% CO_2_, for 48 h. When infection with *B. pertussis* or *B. parapertussis* is suspected, the samples should be inoculated on Regan-Lowe charcoal agar with 10% horse blood and cephalexin, and aerobically incubated under moist conditions at 35 °C, ranging from 5 to 7 days [50]. The specimens potentially containing *N. meningitidis* should be inoculated in Thayer–Martin or another selective medium that supports the growth of such microorganism while inhibiting the proliferation of the microbial population’s normal inhabitants of the upper respiratory airway (5% CO_2_ at 35 °C for 72 h) [50].

A selective medium such as Canada colistin-nalidixic acid, or a selective and differential medium such as BBL CHROMagar *S. aureus* (BD Diagnostics, Sparks, MD), BBL CHROMagar MRSA (BD Diagnostics, Sparks, MD), or mannitol salt agar is helpful in differentiating *S. aureus* or MRSA (methicillin-resistant *S. aureus*) from other bacteria [50].

Regarding LRIs, a qualitative or quantitative (or semiquantitative) culture can be performed. For the qualitative culture of common bacteria, either sputum, BAS, or BAL samples are inoculated on sheep blood agar and MacConkey’s agar (35 °C, 5% CO_2_, 24–48 h); BAL samples can also be cultured under anaerobic conditions on Brucella blood agar, laked blood with kanamycin and vancomycin, and Canada colistin–nalidixic acid [50]. For uncommon bacteria, selective media should be used: *Hemophilus* spp. (chocolate agar, 35 °C, 5% CO_2_, 24–48 h), *Legionella* spp. (buffered charcoal yeast extract with and without antimicrobial agents such as vancomycin, polymyxin B, and anisomycin; aerobic incubation, 35 °C, humidity, 5–10 days), *Chlamydophila* spp. (prompt transport in antibiotic, e.g., gentamycin- and nystatin-containing media for 24–48 h at 4 °C, or for longer periods at −70 °C, and inoculation in shell vials using McCoy cells for *C. trachomatis* and *C. psittaci*, and Hep-2 cells for *C. pneumoniae*), *Burkholderia cepacia* (*B. cepacia* selective agar and oxidative-fermentative-polymyxin B-bacitracin-lactose agar), *M. pneumoniae* (albumin- and penicillin-containing transport medium for up to 24–48 h at 4 °C, or for longer periods at −70 °C and inoculation on mycoplasma–glucose agar, methylene blue–glucose biphasic agar, or SP-4 agar for up to 3 weeks), *S. aureus* (mannitol salt agar), and *Nocardia* spp. (incubation for up to 3 weeks at 35 °C using selective BCYE agar). If the sample is suitable for anaerobic culture, specific media such as Schaedler agar and *Bacteroides* bile esculin agar can be used [50].

However, *Chlamydophila* and *Mycoplasma* species are quite rarely cultured in clinical microbiology laboratories for diagnostic purposes as they require weeks of growth, no easy methods are available, and they result in delayed diagnosis and increased risk of developing severe pneumonia [8]; for these reasons, molecular assays for their detection are preferred.

Quantitative cultures are needed for the diagnosis of VAP, aspiration pneumonia, and pneumonia in immunosuppressed patients or in those with cystic fibrosis. For the BAS specimen, the identification of ≥10^6^ CFU in the original specimen/mL is associated with an active infection; on the contrary, lower counts represent possible cross-contamination. For BAL samples, the recovery of <10^4^ bacteria/mL is most likely to represent contamination, while >10^5^ bacteria/mL is indicative of an active infection. The detection of 10^4^ to 10^5^ bacteria/mL constitutes a “gray zone” [50].

For the detection of the main respiratory viruses (such as ADV, FLU A/B, RSV, and human PIV), the observation and identification of the cytopathic effect in cell culture is considered the gold-standard method [9]. Contextually to RTIs, cell culture is recommended for specific groups of patients, such as immunocompromised patients, children younger than 5 years who complain of respiratory symptoms, and severely ill pediatric patients [9]. Cell culture involves the inoculation of several cell lines with a clinical specimen in an attempt to provide a suitable host for whichever virus might be present on it [9]. The number and the types of cell culture wells are selected based upon the type of clinical specimen, the specimen source, and the supposed causative viral agents [9]. Viral culture wells are then incubated for days to weeks depending on the specimen source and the suspected virus(es) [9]. Cell monolayers are daily screened via microscopic examination to evaluate the potential occurrence of a viral growth [9]. The microscopic examination is performed by placing the plate on the stage of a standard light microscope and viewing the cells through the glass wall of the well with the low-power (10×) objective [9]. The finding of degenerative changes in monolayer cells provides evidence of viral presence [9]. The spectrum of morphological changes ranges from the swelling, shrinking, and rounding of cells to clustering, syncytium formation, and, in some cases, complete destruction of the monolayer. These modifications are collectively called the cytopathogenic or cytopathic effect (CPE) of the virus [9].

Even though the traditional cell culture method is advantageous for growing a wide variety of viruses, including novel or unknown viruses, and it is the only reference laboratory method able to demonstrate viral infectivity, it needs days and often weeks to provide results; thus, it affects patient management and results in poor clinical outcomes [9,39,42].

Over the years, different modified cell culture methods that reduced the turnaround time to 24 h were proposed; even though rapidly modified cell culture methods such as shell vial culture showed similar sensitivity for PIV 1-3 (87% vs. 83%) and influenza A/B (78% vs. 75%), and significantly higher sensitivity for RSV (73% vs. 42%) [56], many clinically relevant viruses are difficult to grow in culture (such as RV and Co-V) and may produce inconclusive results [42]. Moreover, the use and the maintenance of several different cell lines requires technical expertise and makes this method labor-intensive and feasible only in a few specialized centers. Therefore, as compared to molecular assays, the traditional or modified cell culture methods are laborious, exhibit higher false-negative rates, and have longer turnaround times, making viral culture less clinically relevant [42,57,58].

### 4.4. Antigen Detection Assays

Rapid immunoassays are relatively inexpensive, easy to perform, and can deliver test results in less than 30 min; they are commonly named Rapid Diagnostic Test (RDTs). For these reasons, they are invaluable in outpatient clinics, primary care, emergency, and low-resource settings [42,57]. Immunochromatographic assays are considered the most versatile and popular method among the different immunoassays [42].

Currently, for virus detection, commercially available RDTs are mostly limited to FLU A and B virus, and RSV. Despite several studies have demonstrated that RDTs showed overall poor sensitivity for FLU and RSV (44–95%), they have a higher median specificity (90 to 95%) compared to cell culture [42,57], and the sensitivity of RSV immunoassays is relatively higher for children (81%) than adults (29%) [59,60]. During the COVID-19 pandemic, several specific RDTs for the detection of SARS-CoV-2 have been developed and used as point-of-care tests, but their use is specifically limited to the search for this agent in nasal/pharyngeal swabs [61].

With regard to bacteria, such assays allow the prompt detection of the pathogen using respiratory, blood, or urine specimens (mainly for *S. pyogenes*, *S. pneumoniae*, *M. pneumoniae*, *C. pneumoniae*, and *Legionella*). The reported sensitivity in detecting group A *Streptococcus* is 60% to 95% but can be as low as 31% for some assays. Immunochromatographic assays for the detection of the *Legionella* sp. antigen in urine provide a rapid result within 15 min; however, they allow the detection of serogroup 1 only. The urine detection of the polysaccharidic antigen C, present on all pneumococcal serotypes, showed high sensitivity with documented invasive pneumococcal infection; nevertheless, the capability of this method to discriminate between children with true pneumococcal diseases and carriages of rhinopharyngeal diseases is still debated [50].

Depending on their sensitivity and specificity, the use of such assays requires confirmatory assays for a conclusive diagnosis, especially when a negative result is obtained during a respiratory infections season.

### 4.5. Serology

The serologic measurement of specific antibody responses has limited application for the etiologic diagnosis of RTIs, because diagnostic results are only available retrospectively. Efforts have been made to diagnose infections caused by slowly growing or difficult-to-grow microorganisms using serology. This particularly holds for *M. pneumoniae*, *C. pneumoniae*, and *Legionella* infections and viruses. It should be remembered that the most reliable serologic evidence of an ongoing infection is based on a fourfold increase in the titer of IgG (or IgG plus IgM) antibodies during the evolution of the disease episode based on two serum samples collected with an interval of 7 to 10 days or longer, and/or the appearance of IgM antibodies during the evolution of the disease. IgM tests are usually less sensitive and specific than fourfold changes in antibody titers between paired specimens separated by several weeks [62].

Serological tests have been historically performed for the detection of “difficult to isolate” respiratory pathogens, relying either on the detection of IgM in the acute phase of the disease or the demonstration of seroconversion [43].

With regard to viral RTIs, serology allows the identification of antibodies against most of the respiratory pathogens, such as RSV, ADV, FLU A and B, and PIV 1-3 virus, and can detect mixed infections; however, the specific antibodies typically appear about 2 weeks after the initial infection [42,63]. On the other hand, it has been reported that serological assays are significantly less sensitive for the detection of PIV and ADV when compared to molecular methods [42,64]. In general, serum samples for the diagnosis for respiratory infections should be carefully considered; the results of diagnostic assays could be difficult to interpret because of the presence of an immune response to previous exposure to the same agent [50]. In addition, serology is not indicated for immunosuppressed individuals, neonates, or infants because of their impaired immune responses [50].

The serum samples should be collected at least twice during the course of the infection: in the acute phase (as soon as possible after the onset of disease and no later than 1 week) and during convalescence (at least 2 weeks after the clinical manifestation of symptoms). Comparison of the antibody patterns in these two states allows the demonstration of a diagnostically significant active virus, and seroconversion is defined when a fourfold increase in antibody titer occurs [42,50]. In some cases, serologic testing is considered the reference method, such as for Epstein-Barr virus in pharyngitis infection; furthermore, it is also used to check on the effectiveness of vaccinations for specific agents, if available (i.e., FLU and SARS-CoV-2) [50].

As concerns bacterial RTIs, serological testing is crucial for the identification of atypical bacterial agents such as *M. pneumoniae, C. pneumoniae, Legionella* spp., and *B. pertussis.*

In cases of a suspected *M. pneumoniae*-associated RTI, the enzyme immune assay (EIA) is recommended as the reference method to specifically detect IgM or IgG antibodies directed against *M. pneumoniae* [65].

When a *M. pneumoniae*-associated RTI occurs, the specific IgM appear approximately 7 days after the clinical onset, with the peak titers occurring between 4 and 6 weeks after [65]. Since IgM antibodies can persist for 2 months up to 1 year after infection in children, this serological method has been shown to be particularly useful for diagnosis in the pediatric population [65]. As concerns the *C. pneumoniae*-associated RTI, the gold-standard serological method is the microimmunofluorescence (MIF) test, which measures both IgG and IgM antibodies. In particular, the MIF test involves indirect immunofluorescence of the elementary bodies of *C. pneumoniae*, demonstrating high sensitivity if performed with expertise and with properly collected paired sera [66]. The serological diagnosis of *L. pneumophila* can rely on microagglutination, the immunofluorescence assay (IFA), and the enzyme-linked immunosorbent assay (ELISA). These latter two are reported to be excellent techniques in determining the seroprevalence of past and recent infection in a population [67]. The IFA is recommended as the reference method for the diagnosis of *L. pneumophila*-associated RTI, with 75% to 80% sensitivity and >99% specificity when the *L. pneumophila* serotype 1 antigen is used [50]. For the serological diagnosis of *B. pertussis*, the ELISA is the recommended diagnostic method, allowing the detection and the measurement of antibodies directed against the pertussis toxin [68].

However, in this case, the clinical utility of serologic tests is further limited since they require both acute and the convalescent sera to monitor seroconversion and to identify a fourfold increase in antibody titer [42,69]. Different tests showed a range of sensitivity from 14% to 77%, and of specificity from 49% to 97%, compared to PCR [42]. Serology should always be used in combination with confirmatory tests such as those based on direct methods of diagnosis: the isolation and/or acid nucleic detection of specific pathogenic agents.

### 4.6. Nucleic Acid Amplification Tests

Since the early 2000s, several nucleic acid amplification tests for the detection of respiratory pathogens have been commercially available. These tests differ in complexity (i.e., PCR, nucleic acid sequence-based amplification (NASBA), transcription-mediated amplification (TMA), strand displacement amplification (SDA), loop-mediated isothermal amplification (LAMP), rolling circle amplification (RCA), and others) and pathogen coverage; moreover, their accuracy is not only dependent on their specific assay chemistry, but is also critically affected by the type, quantity, and quality of the specimens collected [42].

PCR-based methods for virus detection have been proven to be very sensitive, usually exceeding the sensitivity scores of cell culture techniques. However, false-positive or false-negative results can be a problem if certain measures in handling for the prevention of the viral genetic material are not meticulously followed. Most respiratory viruses have an RNA genome that is particularly vulnerable to degradation by RNAses, which are present in all biologic samples. RNAse-free vials, solutions, and buffers should be used by specialized personnel in designated areas of the laboratory. In addition, if it takes too long for an NPA sample to be transported from the clinic to the laboratory, or if the sample remains on ice for too many hours instead of being frozen immediately, the sensitivity of the method can be unexpectedly low. Further, biologic fluids often contain substances that can inhibit PCR amplification (e.g., mucus). In this case, dilution of the sample or treatment with a suitable agent such as dimethyl sulfoxide may facilitate detection of the virus [50].

Species-specific PCR assays have been developed for numerous bacterial pathogens, with greater accuracy and sensitivity of identification compared to conventional culture-based diagnostics. Despite the fact that nucleic acid persists in specimens after the beginning of therapy and that it may be detected in smaller and noninvasive specimens, this approach requires a prediction to be made as to which is the most likely pathogen, as in the case of selective culture media. Moreover, due to the need for isolation of the microorganism for antibiotic susceptibility testing, cultures have been replaced by molecular methods only in cases in which the pathogens are of predictable susceptibility or the genetics of resistance are well defined, as with MRSA [50]. Assays for the detection of *S. pyogenes* DNA are reported to show a sensitivity of >90%, and by many authors, they are considered sensitive and specific enough to obviate confirmatory culture. Similarly, molecular assays for the detection of *S. aureus* DNA in nasal swabs are as sensitive as the culture but provide faster results [50].

When a prompt diagnosis is urgently required, PCR assays are considered the new gold-standard diagnostic method, as for the detection of *B. pertussis* in rhinopharyngeal samples, or SARS-CoV-2 in nasopharyngeal aspirates [70]. In these cases, PCR assays are significantly more sensitive and specific compared to a culture. In certain cases, such as vaccination, recent contact with an infected individual, sample collection during the paroxysmal stage of the illness, or the administration of antibiotic therapy, the culture is often negative while PCR is positive. Similarly, PCR for the detection of *M. pneumoniae* on rhinopharyngeal aspirates or swabs, or throat swabs, is the most sensitive and specific method, as well as for *C. pneumoniae*, although a positive result may indicate carriage only [50].

It is worth noting that the use of molecular methods for the detection of viral and microbial causative agents of RTIs must be considered only for specific groups of patients complaining of severe clinical respiratory syndromes, such as immunocompromised patients and the pediatric population; it is not recommended for asymptomatic patients or cases of mild infection [9].

## 5. Multiplex Panel Assays

Increasingly advanced molecular diagnostic technologies have the potential to transform and revolutionize microbiological diagnoses in clinical microbiology laboratories, making them faster and more robust [71]. Since 2011, after the first respiratory syndromic panel was cleared by the US Food and Drug Administration (FDA), in less than 10 years, different commercial syndromic panels with different approaches have been introduced; these have expanded the detection of agents that cause infection of the upper and lower respiratory tract (URT/LRT), blood (BL), and gastrointestinal tract (GI), as well as acute meningitis and encephalitis (ME) [72]. The ability to simultaneously detect and identify the most frequent causes of infectious diseases directly from clinical specimens is useful for patient care, hospital infection-control practices, and epidemiologic studies [73]. Respiratory panels comprise various assays that differ in their number and type of pathogens, their qualitative or semi quantitative approach, their manufacture (in-house versus commercial), and their technique (some are point-of-care diagnostic tests). They screen pathogens that infect the upper and/or lower respiratory tract and vary widely in their clinical manifestations [74]. However, for respiratory infections, there is no single generic specimen; nasopharyngeal swabs, sputum, and bronchoalveolar lavage samples are not equivalent. All of these syndromic panels have been constructed according to specimen type [74]. Moreover, the COVID-19 pandemics further highlighted their utility [75], imposing an adaptation of the tests on the new emergency.

Table 2 reports a list of the most relevant FDA-approved syndromic panels for the diagnosis of respiratory illnesses both for the URT and LRT.

Many studies [81,82,83,85,86,87,88,89] have been conducted to evaluate the accuracy of different syndromic panels in specific samples and various patient populations; however, finding enough clinical cases to test could take a long time and more research is needed [79]. As reported, the performances in terms of sensitivity and specificity of these panels are very similar [79], and the greatest number of reported discrepancies between these multiplex panels and reference methods is for ADV and FLU B [79,80]. The formulation of respiratory panels (RPs) not only allows the detection of a broad range of targets, some of which are not detectable otherwise, but also to teaches us about the prevalence and clinical significance of them, such as the demonstration of RV ubiquity and of h-MPV involvement in severe disease [72]. Moreover, this can increase the number of infections that otherwise go undiagnosed because they are not suspected. A recent study demonstrated a 75% higher recovery rate of unexpected *M. pneumoniae* infection using multiple PCR [45]. These results highlight important considerations and limitations of syndromic testing for respiratory tract infections. Among the most important, it should be emphasized that the quantitative values, reported in addition to the qualitative values, suggest caution in interpreting the results to avoid overestimating their significance. In addition, the clinical significance of the detection of multiple agents (a coinfection rate of about 10% was reported) with multiplex panels remains unclear. Many potential clinically relevant microorganisms may be normal flora of URT, particularly if revealed in a lower abundance; as a matter of fact, LRT samples should be evaluated by performing quantitative cultures (i.e., for BAL a concentration higher than 10^4^ CFU in the sample is considered significant). It was reported that for molecular panels, a cutoff of 10^3.5^ genomes/mL is appropriate to consider the detected microorganism as clinically relevant [82]. In any case, analysis of the results should be performed in the context of clinical manifestations, and physicians should interpret both the multiplex PCR result and the final culture results together when establishing antimicrobial therapy plans. Furthermore, it is important to consider inconsistencies with resistance gene detection, especially in cases of co-infections or when the sample is obtained from an anatomical site with low prevalence of resistant pathogens [79]. For example, the CTX-M-type extended-spectrum beta-lactamases gene was reported for any member of the families *Enterobacteriaceae*, *Acinetobacter* spp., or *P. aeruginosa*, and for this reason, when a resistance phenomenon is common to different bacteria, the conventional culture and the phenotypic AST are required to confirm the indication of the resistance marker [82].

The clinical and economic impacts of multiplex respiratory testing have also been evaluated in several studies, concluding that, despite their high cost, multiplex panels offering custom orders can limit unnecessary testing, minimizing patient costs [79]. Different authors demonstrated an improvement in the clinical outcomes of patients after the introduction of RP to the diagnostic workflow caused mainly by the early administration of a targeted antibiotic therapy, and in the rapid adjustment and de-escalation of empirical therapy, also resulting in a short duration of treatment [45,72,75,85]. It was estimated that the multiplex panel results would have allowed for earlier antibiotic adjustment in 70.7% of patients, including de-escalation or discontinuation in 48.2%; this would have resulted in an average of 6.2 antibiotic days saved per patient [85]. In addition to the optimization of antimicrobial use, the application of these tests can reduce hospital admissions and the lengths of stays, as well as the number of chest radiographs and other investigations, as demonstrated by different authors [45]. This is especially true in the COVID-19 era when the potential use of RPs in a setting closer to the patient could be of particular impact in reducing bed moves by 1 day prior to their definitive care area, although the proposed ideal location for RP point-of-care use is the emergency department [85].

The FilmArray^®^ system (BioFireDiagnostics) can identify, in a semi-quantitative mode, both virus- and bacteria-associated pneumonia, as well as determining seven resistance markers (e.g., methicillin- and carbapenem-resistance genes) in 1 h. The extraction, purification of the nucleic acids from the respiratory sample, and nested multiplex PCR are performed in the same cartridge. A dedicated software program automatically analyzes the endpoint melting curve data and reports the detected pathogen [7,76,80,82,83,84].

The Verigene^®^ Respiratory Pathogens Flex Nucleic Acid Test (Nanosphere, Inc.) is performed using the Verigene System, which is a molecular diagnostics workstation consisting of two modules: the Verigene Processor SP and the Verigene Reader. Three automated steps are carried out in the Processor SP: (i) specimen extraction—magnetic bead-based RNA/DNA extraction; (ii) target amplifications; and (iii) hybridization in a microarray format. The Reader can detect, with high efficiency, the target bound in gold–silver aggregates [10,79].

Diagnostic tests with the QIAstat-Dx Respiratory SARS-CoV-2 Panel are performed using the QIAstat-Dx Analyzer 1.0. Samples are collected and loaded manually into the QIAstat-Dx Respiratory SARS-CoV-2 Panel Cartridge, and the extraction, amplification, and detection of nucleic acids in the samples are performed automatically by the QIAstat-Dx Analyzer 1.0. The mixture of the sample and PCR reagents is dispensed into the QIAstat-Dx Respiratory SARS-CoV-2 Panel Cartridge PCR chambers, which contain lyophilized, assay-specific primers and probes. The QIAstat-Dx Analyzer 1.0 creates the optimal temperature profiles to carry out effective multiplex real-time RT-PCR and performs real-time fluorescence measurements to generate amplification curves. The integrated software interprets the resulting data and process controls and delivers a test report [76,77].

The BioCode^®^ MDx-3000 (Applied BioCode, Inc.) is an automated system that integrates PCR amplification, target capture, signal generation and optical detection for multiple respiratory viruses and bacteria. Nucleic acids from NPS are extracted using the BioMérieux NucliSENS^®^ easyMAG^®^ or Roche MagNA Pure 96 automated systems. Once the PCR plate is set up and sealed, all other operations are automated using the MDx-3000. Amplified PCR products labeled with biotin are captured at a defined temperature by target-specific probes that are covalently coupled to designated Barcoded Magnetic Beads (BMBs). High-affinity binding between biotin and streptavidin ensures that captured PCR products with the biotin moiety are labeled with phycoerythrin in close proximity to the BMBs. Optical detection is performed for each reaction well of the capture plate, an optically clear, flat-bottom microtiter plate. Each reaction well is imaged at a specific emission wavelength for its fluorescent signal and under a bright field to identify the barcode patterns (decoding) [78].

The ePlex RP2 Panel (GenMark Diagnostics, Inc.) is an automated qualitative nucleic acid multiplex in vitro diagnostic test for the simultaneous detection and identification of multiple respiratory viral (16 targets) and bacterial (2 targets) nucleic acids. This test is performed using an ePlex instrument that automates all aspects of nucleic acid testing, including extraction, amplification, and detection, combining electrowetting and GenMark’s eSensor^®^ technology in a single-use cartridge. eSensor technology is based on the principles of competitive DNA hybridization and electrochemical detection, which is highly specific and is not based on fluorescent or optical detection [10,79].

The eSensor Respiratory Viral Panel (RVP) (Clinical MicroSensors, Inc.) is a qualitative nucleic acid multiplex test intended for use on the eSensor XT-8 system for the simultaneous detection and identification of multiple respiratory viral nucleic acids. The eSensor XT-8 consumable has a plurality of electrode locations that are coated with analyte-specific capture probe oligonucleotide for multiplex amplicon detection. The eSensor XT-8 System accepts the consumable and completes the hybridization and detection of each electrode using an assay-specific protocol [80].

The Luminex NxTAG^®^ Respiratory Pathogen Panel–(RPP)–CE-IVD is a qualitative nucleic acid multiplex test that provides simultaneous detection and identification of 18 viruses and 3 atypical bacteria associated with RTIs. The NxTAG Respiratory Pathogen Panel is a ready-to-use system requiring very little hands-on time and is performed in a closed PCR vessel, reducing the chances of contamination. Nucleic acid is simply added directly to pre-plated lyophilized reagents for RT-PCR and bead hybridization. The results are read on the MAGPIX^®^ instrument; then, the data are analyzed with the RPP assay-specific Software Accessory Package using SYNCT™ software [10,79,80].

## 6. Conclusions

This review focuses on the technologies used at present for the laboratory diagnosis of infectious respiratory diseases, showing that no single approach, whether it is molecular detection, antigen identification, or virus/bacteria isolation, meets the needs of all diagnostic microbiology/virology laboratories in all clinical situations involving all types of bacteria/viruses. Clinical microbiologists and virologists are challenged to use the available technology that best fits the particular situation and yields the most useful results, and should produce clinical reports that are able to guide physicians toward the right interpretation of the results for the best management of the patient.

Tomorrow, as more sophisticated, yet simpler-to-use, broad-range molecular platforms become available for clinical diagnostics, bacteria cultivation and/or virus isolation in cell culture may once again become mainly a research tool. Therefore, culture- and the non-culture-based methods should be performed in parallel to optimize the differential diagnosis of viral and microbial diseases, in order to obtain useful, cost-effective, and labor-saving microbial and/or viral testing results. In determining appropriate testing algorithms for the laboratory, laboratorians must consider a wide range of factors, including the patient population (i.e., age, immune status, and comorbidities), the clinical manifestations, the physician’s diagnosis, the changing epidemiology, and time of year (i.e., many viral infections tend to be seasonal).

Among the advantages and disadvantages, the cost of the molecular assays compared to that of conventional assays should be taken into account. Considering the cost per assay, syndromic panels are expensive at about EUR 100–200 per sample, allowing the detection of 14 to 27 agents per run, according to the assay. On the contrary, the culture-based assays, including MALDI-ToF identification and AST, cost about 30 Euros per sample, only allowing the detection of viable agents or cultivable agents (viruses are not yet cultivable and fastidious microorganisms are not included).

The current algorithms for the diagnosis of RTIs include multiple approaches, among which molecular methods and conventional culture are the most used for laboratory diagnosis of such infectious diseases. Molecular methods are the most used for the detection of viral agents and many atypical bacteria, and their use should be routinely applied in clinical laboratories to samples from patients in the emergency department. The conventional culture remains the gold-standard for the detection of bacteria but suffers from several shortcomings. In particular, culture-based methods show lower sensitivity than molecular methods, particularly with regard to the detection of “difficult-to-grow” microbes, thus underestimating viable microorganisms in the sample to be tested. Moreover, a conventional culture is time-consuming since it requires an average of 48 to 72 h for time-to-results.

Specimen-processing guidelines vary from laboratory to laboratory, resulting in the lack of a common line in the interpretation of growth bacterial patterns, with different modes of reporting the results. On the other hand, the gold-standard cell culture for the viral diagnosis of RTIs also shows several disadvantages: the need for technical expertise in evaluating the cell culture monolayers, the long incubation period required for some viruses to produce CPE, the inability of some viruses to proliferate in traditional cell cultures, and the expense involved in purchasing and maintaining cell cultures are all factors to consider when evaluating such as diagnostic workflow.

The implementation of syndromic panels in the respiratory infection diagnostic algorithm has the potential to be a powerful decision-making tool for patient management, especially in emergency departments, despite requiring the appropriate use of the test in different patient populations. It is mandatory that their use is limited to symptomatic subjects, immunocompromised patients, children less than 5 years old, and the elderly, and that their use is avoided in asymptomatic subjects or mild infections.

In conclusion, the use of syndromic panels for the detection of respiratory pathogens is associated with a radically reduced time-to-results and, in parallel, to increased detection of clinically relevant pathogens compared to the standard methods. Syndromic panels, if implemented wisely and interpreted cautiously, can improve antimicrobial use and patient outcomes through improved clinical decision, optimized laboratory workflow, and enhanced antimicrobial and laboratory stewardship. As the implementation of new syndromic diagnostic platforms in clinical diagnosis continues to grow, it will be essential to share experiences regarding implementation and optimization strategies. Further research is therefore needed to understand the relationship between the number of viruses/bacteria and its clinical relevance in different patient populations, as well as the true clinical significance of the simultaneous finding of multiple pathogens.

## Figures and Tables

**Table 1:** The main etiological agents of pneumonia.

Bacteria	Viruses	Fungi
*Streptococcus pneumoniae* *Staphylococcus aureus* *Haemophylus influenzae* type b *Klebsiella pneumoniae* *Pseudomonas aeruginosa* *Chlamydophila pneumoniae* *Mycoplasma pneumoniae* *Legionella pneumophyla * *Chlamydia trachomatis* *Chlamydophila psittaci* *Coxiella burnetii*	Influenza virus A Parainfluenza virus 1, 2, 3 Respiratory syncytial virus Adenovirus 1–7, 14, 21 SARS-CoV-1, 2 * MERS ** Epstein-Barr virus Coxsackie A virus Cytomegalovirus	*Histoplasma capsulatum* *Coccidioides immitis* *Blastomyces brasiliensis* *Aspergillus* spp. *Candida* spp. *Cryptococcus neoformans* *Pneumocystis jirovecii*

Abbreviations: * SARS-CoV: Severe Acute Respiratory Syndrome Coronavirus. ** MERS: Middle East Respiratory Syndrome.

**Table 2:** The most relevant FDA-approved syndromic panels for the diagnosis of respiratory illnesses for both the upper and the lower respiratory tracts.

Assay	Company	Target	Time-to-Result	Type of Amplification	Reference
QIAstat-Dx Respiratory SARS-CoV-2 Panel	QIAGEN GmbH	22 Pathogens (FLU A, FLU A subtype H1N1/2009, H1, and H3; FLU B; CoV 229E, HKU1, NL63, OC43; SARS-CoV-2; PIV 1, 2, 3, and 4; RSV A/B; h-MPV A/B; ADV; h-BocaV; RV/Enterovirus; *Mycoplasma pneumoniae*; *Legionella pneumophila*; *Bordetella pertussis*)	About 1 h	Multiplex real-time RT-PCR	[76,77]
BioCode Respiratory Pathogen Panel (RPP)	Applied BioCode, Inc.	17 Pathogens (ADV; CoV 229E, OC43, HKU1, NL63; h-MPV A/B; FLU A, FLU A subtype H1N1/2009, H1, and H3; FLU B; PIV 1, 2, 3, and 4; RSV A/B; RV/Enterovirus; *Bordetella pertussis*; *Chlamydia pneumoniae*; *Mycoplasma pneumoniae*)	About 5 h	RT-PCR	[78]
ePlex Respiratory Pathogen Panel 2	GenMarkDiagnostics, Inc.	18 Pathogens (ADV; CoV 229E, OC43, HKU1, NL63; SARS-CoV-2; h-MPV A/B; FLU A, FLU A subtype H1N1/2009, H1, and H3; FLU B, PIV 1, 2, 3, and 4; RSV A, B; RV/Enterovirus; *Chlamydia pneumoniae*; *Mycoplasma pneumoniae*)	About 2 h	RT-PCR	[10,79]
eSensor Respiratory Viral Panel (RVP)	Clinical MicroSensors, Inc.	14 Pathogens (FLU A, FLU A subtype H1N1/2009, H1, and H3; FLU B; RSV A, B; PIV 1, 2, and 3; h-MPV; RV; ADV species B/E; ADV species C)	About 8 h	Multiplex microarray, competitive DNA hybridization	[80]
FilmArray Pneumonia plus Panel	BioFireDiagnostics, LLC	27 Pathogens and 7 resistant genes (Semi-quantitative detection: *Acinetobacter calcoaceticus-baumannii complex*; *Enterobacter cloacae*; *Escherichia coli*; *Haemophilus influenzae*; *Klebsiella aerogenes*; *Klebsiella oxytoca*; *Klebsiella pneumoniae* group; *Moraxella catarrhalis*; *Proteus* spp.; *Pseudomonas aeruginosa*; *Serratia marcescens*; *Staphylococcus aureus*; *Streptococcus agalactiae*; *Streptococcus pneumoniae*; *Streptococcus pyogenes* Qualitative detection: *Legionella pneumophila*; *Mycoplasma pneumoniae*; *Chlamydia pneumoniae*; FLU A; FLU B; ADV; CoV; PIV; RSV; RV/Enterovirus; h-MPV; Middle East Respiratory Syndrome Coronavirus Resistance: ESBL: CTX-M; Carbapenemases: KPC, NDM, Oxa48-like, VIM, IMP; Methicilin Resistance: mecA/mecC and MREJ)	About 1 h	Nested multiplex RT-PCR	[7,81,82,83]
FilmArray Respiratory Panel (RP)	BioFireDiagnostics, LLC	20 Pathogens (ADV; CoV 229E, HKU1, OC43, NL63; h-MPV; RV/Enterovirus; FLU A, FLU A subtype H1N1/2009, H1, and H3; FLU B; PIV 1, 2, 3, and 4; RSV; *Bordetella pertussis*; *Chlamydophila pneumoniae*; *Mycoplasma pneumoniae*)	About 1 h	Nested multiplex RT-PCR	[80]
NxTAG Respiratory Pathogen Panel	LuminexMolecularDiagnostics, Inc.	22 Pathogens (FLU A, FLU A subtype H1N1/2009, H1, and H3; FLU B; RSV A, B; CoV 229E, OC43, NL63, HKU1; PIV 1, 2, 3, and 4; h-MPV; ADV; h-BocaV; RV/Enterovirus; *Chlamydophila pneumoniae*; *Mycoplasma pneumoniae*; *Legionella pneumophyla*)	About 4 h	RT-PCR	[10,79,80]
xTAG Respiratory Viral Panel Fast (RVP FAST)	LuminexMolecularDiagnostics, Inc.	18 Pathogens (FLU A, FLU A subtype H1 and H3; FLU B; RSV A, B; CoV 229E, OC43, NL63, HKU1; PIV 1, 2, 3, and 4; h-MPV; ADV; h-BocaV; RV/Enterovirus)	About 4 h	RT-PCR	[10]
Verigene Respiratory Pathogens Flex NucleicAcid Test (RP Flex)	Nanosphere, Inc.	16 Pathogens (ADV; h-MPV; FLU A, FLU A subtype H1 and H3; FLU B; PIV 1, 2, 3, and 4; RV; RSV A, B; *Bordetella pertussis*; *Bordetella parapertussis/bronchiseptica*; *Bordetella holmesii*)	About 2 h	RT-PCR and microarray hybridization	[10,79]
FilmArray Respiratory Panel 2.1 (RP 2.1)	BioFireDiagnostics, LLC	19 Pathogens (ADV; CoV 229E, HKU1, OC43, NL63; SARS-CoV-2; h-MPV; RV/Enterovirus; FLU A, FLU A subtype H1N1/2009, H1, and H3; FLU B; PIV; RSV; *Bordetella pertussis*; *Bordetella parapertussis*; *Chlamydophila pneumoniae*; *Mycoplasma pneumoniae*)	About 1 h	Nested multiplex RT-PCR	[76,84]
FilmArray Respiratory Panel 2.1 plus (RP2PLUS)	BioFireDiagnostics, LLC	21 Pathogens (ADV; CoV 229E, HKU1, OC43, NL63; Middle East Respiratory Syndrome Coronavirus; SARS-CoV-2; h-MPV; RV/Enterovirus; FLU A, FLU A subtype H1N1/2009, H1, and H3; FLU B; PIV 1, 2, 3, and 4; RSV; *Bordetella pertussis*; *Bordetella parapertussis*; *Chlamydophila pneumoniae*; *Mycoplasma pneumoniae*)	About 45 min	Nested multiplex RT-PCR	[84]

## Data Availability

The data presented in this study are available in the manuscript.

## References

[1] WHO Severe Acute Respiratory Infections Treatment Centre (2020). Practical Manual to Set Up and Manage a SARI Treatment Centre and a SARI Screening Facility in Health Care Facilities.

[2] WHO (2014). Infection Prevention and Control of Epidemic- and Pandemic-Prone Acute Respiratory Infections in Health Care: WHO Guidelines.

[3] Mirsaeidi M., Motahari H., Taghizadeh Khamesi M., Sharifi A., Campos M., Schraufnagel D.E. (2016). Climate Change and Respiratory Infections. Ann. Am. Thorac. Soc..

[4] Huang G., Guo F. (2022). Loss of Life Expectancy Due to Respiratory Infectious Diseases: Findings from the Global Burden of Disease Study in 195 Countries and Territories 1990–2017. J. Popul. Res..

[5] Yanagihara K. (2019). The Role of Molecular Diagnosis in Acute Respiratory Tract Infection. Respir. Investig..

[6] Kuti E.L., Patel A.A., Coleman C.I. (2008). Impact of Inappropriate Antibiotic Therapy on Mortality in Patients with Ventilator-Associated Pneumonia and Blood Stream Infection: A Meta-Analysis. J. Crit. Care.

[7] Webber D.M., Wallace M.A., Burnham C.A., Anderson N.W. (2020). Evaluation of the BioFire FilmArray Pneumonia Panel for Detection of Viral and Bacterial Pathogens in Lower Respiratory Tract Specimens in the Setting of a Tertiary Care Academic Medical Center. J. Clin. Microbiol..

[8] Rytter H., Jamet A., Coureuil M., Charbit A., Ramond E. (2020). Which Current and Novel Diagnostic Avenues for Bacterial Respiratory Diseases?. Front. Microbiol..

[9] Leland D.S., Ginocchio C.C. (2007). Role of Cell Culture for Virus Detection in the Age of Technology. Clin. Microbiol. Rev..

[10] Couturier M.R., Bard J.D. (2019). Direct-from-Specimen Pathogen Identification: Evolution of Syndromic Panels. Clin. Lab. Med..

[11] WHO The Global Health Observatory Global Health Estimates: Leading Causes of Death. Cause-Specific Mortality, 2000–2019. https://www.who.int/data/gho/data/themes/mortality-and-global-health-estimates/ghe-leading-causes-of-death.

[12] WHO The Global Health Observatory Global Health Estimates: Leading Causes of DALYs. Disease Burden, 2000–2019. https://www.who.int/data/gho/data/themes/mortality-and-global-health-estimates/global-health-estimates-leading-causes-of-dalys.

[13] The World Bank World Bank Country and Lending Groups Country Classification. https://datahelpdesk.worldbank.org/knowledgebase/articles/906519-world-bank-country-and-lending-groups.

[14] Simoes E.A.F., Cherian T., Chow J., Shahid-Salles S.A., Laxminarayan R., John T.J., Jamison D.T., Breman J.G., Measham A.R., Alleyne G., Claeson M., Evans D.B., Jha P., Mills A., Musgrove P. (2006). Acute Respiratory Infections in Children.

[15] WHO International Statistical Classification of Diseases and Related Health Problems (ICD). https://www.who.int/standards/classifications/classification-of-diseases.

[16] Grief S.N. (2013). Upper Respiratory Infections. Prim. Care.

[17] Miller J.M., Binnicker M.J., Campbell S., Carroll K.C., Chapin K.C., Gilligan P.H., Gonzalez M.D., Jerris R.C., Kehl S.C., Patel R. (2018). A Guide to Utilization of the Microbiology Laboratory for Diagnosis of Infectious Diseases: 2018 Update by the Infectious Diseases Society of America and the American Society for Microbiology. Clin. Infect. Dis. Off. Publ. Infect. Dis. Soc. Am..

[18] Flores A.R., Caserta M.T. (2015). Pharyngitis. Mandell, Douglas, and Bennett’s Principles and Practice of Infectious Diseases.

[19] Arnold J.C., Nizet V. (2018). Pharyngitis. Principles and Practice of Pediatric Infectious Diseases.

[20] Mustafa Z., Ghaffari M. (2020). Diagnostic Methods, Clinical Guidelines, and Antibiotic Treatment for Group A Streptococcal Pharyngitis: A Narrative Review. Front. Cell. Infect. Microbiol..

[21] Wolford R.W., Goyal A., Belgam Syed S.Y., Schaefer T.J. (2022). Pharyngitis.

[22] Alcaide M.L., Bisno A.L. (2007). Pharyngitis and Epiglottitis. Infect. Dis. Clin. N. Am..

[23] Weber R. (2014). Pharyngitis. Prim. Care.

[24] Anderson J., Paterek E. (2022). Tonsillitis.

[25] Bartlett A., Bola S., Williams R. (2015). Acute Tonsillitis and Its Complications: An Overview. J. R. Nav. Med. Serv..

[26] Tristram D. (2018). Laryngitis, Tracheitis, Epiglottitis, and Bronchiolitis: Sore Throat, Change in Voice, Feverora Wheezing Infant in Respiratory Distress. Introduction to Clinical Infectious Diseases: A Problem-Based Approach.

[27] Caserta M.T. (2015). Acute Laryngitis. Mandell, Douglas, and Bennett’s Principles and Practice of Infectious Diseases.

[28] Blot M., Bonniaud-Blot P., Favrolt N., Bonniaud P., Chavanet P., Piroth L. (2017). Update on Childhood and Adult Infectious Tracheitis. Med. Mal. Infect..

[29] Bower J., McBride J.T. (2015). Croup in Children (Acute Laryngotracheobronchitis). Mandell, Douglas, and Bennett’s Principles and Practice of Infectious Diseases.

[30] Woodfork K. (2007). Bronchitis. xPharm: The Comprehensive Pharmacology Reference.

[31] Walsh E.E. (2015). Acute Bronchitis. Mandell, Douglas, and Bennett’s Principles and Practice of Infectious Diseases.

[32] de Conto F., Conversano F., Medici M.C., Ferraglia F., Pinardi F., Arcangeletti M.C., Chezzi C., Calderaro A. (2019). Epidemiology of Human Respiratory Viruses in Children with Acute Respiratory Tract Infection in a 3-Year Hospital-Based Survey in Northern Italy. Diagn. Microbiol. Infect. Dis..

[33] Rossi G.A., Medici M.C., Merolla R. (2005). Incidence of Respiratory Syncytial Virus Positivity in Young Italian Children Referred to the Emergency Departments for Lower Respiratory Tract Infection over Two Consecutive Epidemic Seasons. Infection.

[34] Segal L.N., Weiden M.D., Horowitz H.W. (2015). Acute Exacerbations of Chronic Obstructive Pulmonary Disease. Mandell, Douglas, and Bennett’s Principles and Practice of Infectious Diseases.

[35] Medici M.C., Arcangeletti M.C., Merolla R., Chezzi C. (2004). Incidence of Respiratory Syncytial Virus Infection in Infants and Young Children Referred to the Emergency Departments for Lower Respiratory Tract Diseases in Italy. Acta Biomed..

[36] WHO Pneumonia. https://www.who.int/news-room/fact-sheets/detail/pneumonia.

[37] Fauci A.S. (2015). Harrison’s Principles of Internal Medicine.

[38] Lanks C.W., Musani A.I., Hsia D.W. (2019). Community-Acquired Pneumonia and Hospital-Acquired Pneumonia. Med. Clin. N. Am..

[39] Calderaro A., Buttrini M., Montecchini S., Piccolo G., Martinelli M., Dell’Anna M.L., di Maio A., Arcangeletti M.C., Maccari C., de Conto F. (2021). Detection of SARS-CoV-2 and Other Infectious Agents in Lower Respiratory Tract Samples Belonging to Patients Admitted to Intensive Care Units of a Tertiary-Care Hospital, Located in an Epidemic Area, during the Italian Lockdown. Microorganisms.

[40] Weinberger S.E., Cockrill B.A., Mandel J. (2017). Principles of Pulmonary Medicine E-Book.

[41] Albano D., Bertagna F., Alongi P., Baldari S., Baldoncini A., Bartolomei M., Boccaletto F., Boero M., Borsatti E., Bruno A. (2021). Prevalence of Interstitial Pneumonia Suggestive of COVID-19 at (18)F-FDG PET/CT in Oncological Asymptomatic Patients in a High Prevalence Country during Pandemic Period: A National Multi-Centric Retrospective Study. Eur. J. Nucl. Med. Mol. Imaging.

[42] Das S., Dunbar S., Tang Y.-W. (2018). Laboratory Diagnosis of Respiratory Tract Infections in Children-the State of the Art. Front. Microbiol..

[43] Murdoch D.R., Werno A.M., Jennings L.C. (2019). Microbiological Diagnosis of Respiratory Illness: Recent Advances. Kendig’s Disorders of the Respiratory Tract in Children.

[44] Campbell S., Forbes B.A. (2011). The Clinical Microbiology Laboratory in the Diagnosis of Lower Respiratory Tract Infections. J. Clin. Microbiol..

[45] Ramanan P., Bryson A.L., Binnicker M.J., Pritt B.S., Patel R. (2018). Syndromic Panel-Based Testing in Clinical Microbiology. Clin. Microbiol. Rev..

[46] Leber A.L. (2020). Clinical Microbiology Procedures Handbook.

[47] Centers for Disease Control and Prevention (2020). Specimen Collection Guidelines.

[48] Charalambous B.M., Batt S.L., Peek A.C., Mwerinde H., Sam N., Gillespie S.H. (2003). Quantitative Validation of Media for Transportation and Storage of Streptococcus Pneumoniae. J. Clin. Microbiol..

[49] Poulsen C.S., Kaas R.S., Aarestrup F.M., Pamp S.J. (2021). Standard Sample Storage Conditions Have an Impact on Inferred Microbiome Composition and Antimicrobial Resistance Patterns. Microbiol. Spectr..

[50] Skevaki C.L., Papadopoulos N.G., Tsakris A., Johnston S.L. (2012). Microbiologic Diagnosis of Respiratory Illness: Practical Applications. Kendig & Chernick’s Disorders of the Respiratory Tract in Children.

[51] Cooley L., Dendle C., Wolf J., Teh B.W., Chen S.C., Boutlis C., Thursky K.A. (2014). Consensus Guidelines for Diagnosis, Prophylaxis and Management of *P Neumocystis Jirovecii* Pneumonia in Patients with Haematological and Solid Malignancies, 2014. Intern. Med. J..

[52] Falsey A.R., Walsh E.E. (2003). Novel Coronavirus and Severe Acute Respiratory Syndrome. Lancet.

[53] Ksiazek T.G., Erdman D., Goldsmith C.S., Zaki S.R., Peret T., Emery S., Tong S., Urbani C., Comer J.A., Lim W. (2003). A Novel Coronavirus Associated with Severe Acute Respiratory Syndrome. N. Engl. J. Med..

[54] Goldsmith C.S., Miller S.E. (2009). Modern Uses of Electron Microscopy for Detection of Viruses. Clin. Microbiol. Rev..

[55] Zhang Y., Hung T., Song J., He J. (2013). Electron Microscopy: Essentials for Viral Structure, Morphogenesis and Rapid Diagnosis. Sci. China Life Sci..

[56] LaSala P.R., Bufton K.K., Ismail N., Smith M.B. (2007). Prospective Comparison of R-Mix Shell Vial System with Direct Antigen Tests and Conventional Cell Culture for Respiratory Virus Detection. J. Clin. Virol. Off. Publ. Pan Am. Soc. Clin. Virol..

[57] Ginocchio C.C. (2007). Detection of Respiratory Viruses Using Non-Molecular Based Methods. J. Clin. Virol. Off. Publ. Pan Am. Soc. Clin. Virol..

[58] Hematian A., Sadeghifard N., Mohebi R., Taherikalani M., Nasrolahi A., Amraei M., Ghafourian S. (2016). Traditional and Modern Cell Culture in Virus Diagnosis. Osong Public Health Res. Perspect..

[59] Slinger R., Milk R., Gaboury I., Diaz-Mitoma F. (2004). Evaluation of the QuickLab RSV Test, a New Rapid Lateral-Flow Immunoassay for Detection of Respiratory Syncytial Virus Antigen. J. Clin. Microbiol..

[60] Chartrand C., Tremblay N., Renaud C., Papenburg J. (2015). Diagnostic Accuracy of Rapid Antigen Detection Tests for Respiratory Syncytial Virus Infection: Systematic Review and Meta-Analysis. J. Clin. Microbiol..

[61] WHO (2021). Antigen-Detection in the Diagnosis of SARS-CoV-2 Infection.

[62] Loens K., van Heirstraeten L., Malhotra-Kumar S., Goossens H., Ieven M. (2009). Optimal Sampling Sites and Methods for Detection of Pathogens Possibly Causing Community-Acquired Lower Respiratory Tract Infections. J. Clin. Microbiol..

[63] Chkhaidze I., Manjavidze N., Nemsadze K. (2006). Serodiagnosis of Acute Respiratory Infections in Children in Georgia. Indian J. Pediatr..

[64] Kuypers J., Wright N., Ferrenberg J., Huang M.-L., Cent A., Corey L., Morrow R. (2006). Comparison of Real-Time PCR Assays with Fluorescent-Antibody Assays for Diagnosis of Respiratory Virus Infections in Children. J. Clin. Microbiol..

[65] File T.M.J., Tan J.S., Plouffe J.F. (1998). The Role of Atypical Pathogens: Mycoplasma Pneumoniae, Chlamydia Pneumoniae, and Legionella Pneumophila in Respiratory Infection. Infect. Dis. Clin. N. Am..

[66] Saikku P. (1998). Diagnosis of Chlamydia Pneumoniae. Clin. Microbiol. Infect. Off. Publ. Eur. Soc. Clin. Microbiol. Infect. Dis..

[67] Graham F.F., Hales S., White P.S., Baker M.G. (2020). Review Global Seroprevalence of Legionellosis—A Systematic Review and Meta-Analysis. Sci. Rep..

[68] WHO (2014). Laboratory Manual for the Diagnosis of Wooping Cough Caused by Bordetella Pertussis/Bordetella Parapertussis.

[69] Loeffelholz M., Chonmaitree T. (2010). Advances in Diagnosis of Respiratory Virus Infections. Int. J. Microbiol..

[70] WHO (2022). Clinical Care for Severe Acute Respiratory Infection.

[71] Messacar K., Parker S.K., Todd J.K., Dominguez S.R. (2017). Implementation of Rapid Molecular Infectious Disease Diagnostics: The Role of Diagnostic and Antimicrobial Stewardship. J. Clin. Microbiol..

[72] Dien Bard J., McElvania E. (2020). Panels and Syndromic Testing in Clinical Microbiology. Clin. Lab. Med..

[73] Hanson K.E., Couturier M.R. (2016). Multiplexed Molecular Diagnostics for Respiratory, Gastrointestinal, and Central Nervous System Infections. Clin. Infect. Dis. Off. Publ. Infect. Dis. Soc. Am..

[74] Zanella M.-C., Meylan P., Kaiser L. (2020). Syndromic Panels or “Panel Syndrome”? A Perspective through the Lens of Respiratory Tract Infections. Clin. Microbiol. Infect. Off. Publ. Eur. Soc. Clin. Microbiol. Infect. Dis..

[75] Fox A.S., Rao S.N. (2021). Syndromic Testing for the Diagnosis of Infectious Diseases: The Right Test If Used for the Right Patient. J. Antimicrob. Chemother..

[76] Cassidy H., van Genne M., Lizarazo-Forero E., Niesters H.G.M., Gard L. (2022). Evaluation of the QIAstat-Dx RP2.0 and the BioFire FilmArray RP2.1 for the Rapid Detection of Respiratory Pathogens Including SARS-CoV-2. Front. Microbiol..

[77] Lebourgeois S., Storto A., Gout B., le Hingrat Q., Ardila Tjader G., del Carmen Cerdan M., English A., Pareja J., Love J., Houhou-Fidouh N. (2021). Performance Evaluation of the QIAstat-Dx^®^ Respiratory SARS-CoV-2 Panel. Int. J. Infect. Dis..

[78] Yu C.Y., Chan K.G., Yean C.Y., Ang G.Y. (2021). Nucleic Acid-Based Diagnostic Tests for the Detection SARS-CoV-2: An Update. Diagnostics.

[79] Cassidy H., van Genne M., Lizarazo-Forero E., Gard L., Niesters H.G.M. (2021). A Discussion of Syndromic Molecular Testing for Clinical Care. J. Antimicrob. Chemother..

[80] Popowitch E.B., O’Neill S.S., Miller M.B. (2013). Comparison of the Biofire FilmArray RP, Genmark ESensor RVP, Luminex XTAG RVPv1, and Luminex XTAG RVP Fast Multiplex Assays for Detection of Respiratory Viruses. J. Clin. Microbiol..

[81] Lee S.H., Ruan S.-Y., Pan S.-C., Lee T.-F., Chien J.-Y., Hsueh P.-R. (2019). Performance of a Multiplex PCR Pneumonia Panel for the Identification of Respiratory Pathogens and the Main Determinants of Resistance from the Lower Respiratory Tract Specimens of Adult Patients in Intensive Care Units. J. Microbiol. Immunol. Infect..

[82] Murphy C.N., Fowler R., Balada-Llasat J.M., Carroll A., Stone H., Akerele O., Buchan B., Windham S., Hopp A., Ronen S. (2020). Multicenter Evaluation of the BioFire FilmArray Pneumonia/Pneumonia Plus Panel for Detection and Quantification of Agents of Lower Respiratory Tract Infection. J. Clin. Microbiol..

[83] Buchan B.W., Windham S., Balada-Llasat J.-M., Leber A., Harrington A., Relich R., Murphy C., Dien Bard J., Naccache S., Ronen S. (2020). Practical Comparison of the BioFire FilmArray Pneumonia Panel to Routine Diagnostic Methods and Potential Impact on Antimicrobial Stewardship in Adult Hospitalized Patients with Lower Respiratory Tract Infections. J. Clin. Microbiol..

[84] Tazi S., Kabbaj H., Zirar J., Zouaki A., el Amin G., el Himeur O., Seffar M. (2022). Comparative Performance Evaluation of FilmArray BioFire RP2.1 and MAScIR 2.0 Assays for SARS-CoV-2 Detection. Adv. Virol..

[85] Dumkow L.E., Worden L.J., Rao S.N. (2021). Syndromic Diagnostic Testing: A New Way to Approach Patient Care in the Treatment of Infectious Diseases. J. Antimicrob. Chemother..

[86] Rappo U., Schuetz A.N., Jenkins S.G., Calfee D.P., Walsh T.J., Wells M.T., Hollenberg J.P., Glesby M.J. (2016). Impact of Early Detection of Respiratory Viruses by Multiplex PCR Assay on Clinical Outcomes in Adult Patients. J. Clin. Microbiol..

[87] Rogers B.B., Shankar P., Jerris R.C., Kotzbauer D., Anderson E.J., Watson J.R., O’Brien L.A., Uwindatwa F., McNamara K., Bost J.E. (2015). Impact of a Rapid Respiratory Panel Test on Patient Outcomes. Arch. Pathol. Lab. Med..

[88] Srinivas P., Rivard K.R., Pallotta A.M., Athans V., Martinez K., Loutzenheiser S., Lam S.W., Procop G.W., Richter S.S., Neuner E.A. (2019). Implementation of a Stewardship Initiative on Respiratory Viral PCR-based Antibiotic Deescalation. Pharmacother. J. Hum. Pharmacol. Drug Ther..

[89] Brendish N.J., Mills S., Ewings S., Clark T.W. (2019). Impact of Point-of-Care Testing for Respiratory Viruses on Antibiotic Use in Adults with Exacerbation of Airways Disease. J. Infect..

